# Supplementary material for: MetaRibo-Seq measures translation in microbiomes
Source: Nat Commun. 2020 Jun 29;11:3268. doi: 10.1038/s41467-020-17081-z (PMC7324362; doi:10.1038/s41467-020-17081-z)
Supplement: Supplementary file 10 — Supplementary Data 7 [file 41467_2020_17081_MOESM10_ESM.zip › File2/Confidence_High_Taxonomy/357987_out.krona.html]

Javascript must be enabled to view this page.

members
magnitude
magnitudeUnassigned
count
unassigned
taxon
rank

357987\_out

1
5

SRS051031\_contig\_number\_27193

1
2
superkingdom

976
phylum
1

class
200643
1

171549
order
1


SRS023914\_contig\_number\_22482
1
species
1674831

3
2759
superkingdom

4751
kingdom
3

451864
subkingdom
3

3
4890
phylum

subphylum
147537
1

class
4891
1


SRS053356\_contig\_number\_27594
1
order
4892

147538
subphylum
2

2
147541
class

2
451867
subclass

order
134362
2

genus
766053
2

species
766055
2

SRS013687\_contig\_number\_28251SRS098571\_contig\_number\_57243
